# Cognitive training for older prisoners: a qualitative analysis of prisoners’ and staff members’ perceptions

**DOI:** 10.3389/fnagi.2023.1332136

**Published:** 2024-01-08

**Authors:** Sandra Verhülsdonk, Ann-Kristin Folkerts, Caroline Hasenberg, Claire Bohn, Julia Christl, Elke Kalbe, Theresia Krieger

**Affiliations:** ^1^Department of Psychiatry and Psychotherapy, LVR-Klinikum Düsseldorf, Medical Faculty, Heinrich Heine University Düsseldorf, Düsseldorf, Germany; ^2^Medical Psychology, Neuropsychology and Gender Studies, Faculty of Medicine and University Hospital Cologne, University of Cologne, Cologne, Germany

**Keywords:** correctional facilities, older prisoners, cognitive training, cognitive dysfunction, cognition

## Abstract

**Introduction:**

Correctional institutions are challenged by increasing numbers of older prisoners. Existing literature highlights the vulnerability of this group that is reflected by various somatic and mental health issues including cognitive dysfunctions. Although cognitive training studies in various target groups of older people have been conducted, there is lack of data regarding cognitive training in older prisoners. A structured cognitive group training program (“NEUROvitalis Prison”) with 12 weekly sessions was offered to male prisoners in Germany.

**Methods:**

Post intervention an exploratory qualitative study was conducted. Prisoners (*N* = 18) and staff (*N* = 4) perspectives were explored by conducting face-to-face semi-structured interviews. Audiotaped data were fully transcribed and deductive-inductive content analyses applied.

**Results:**

Both the prisoners and the staff perceived the cognitive training as very positive and stimulating. Moreover, the importance of the training was pronounced in terms of an increase in self-esteem and understanding of cognition and aging in the prisoners.

**Discussion:**

Our data indicate that cognitive training may be a feasible and valuable intervention for older prisoners that will be appreciated by both inmates and staff. The qualitative data provide substantial insight into the experiences with the applied cognitive training program. Moreover, valuable modifications for future conduct can be derived.

## 1 Introduction

### 1.1 Background

In line with the general demographic change, the number of older people within the prison system also increases significantly in industrialized countries. For example, in Germany, from 1993 to 2014, the number of prisoners over the age of 60 increased from 506 to 2,246. Nevertheless, little data is available on the extent to which this increase is also associated with an increase in age-typical diseases. Notably, a survey by Meyer (2016) points to poorer health (as measured by various diagnoses) and more pronounced functional deficits among older prisoners compared to an extramural control group. In addition, chronic illness as well as cognitive impairment are common problems among this group (Canada et al., 2019). A review of surveys in the United States, Canada, England, and Wales by Kakoullis et al. (2010) describes the prevalence for cognitive impairment among older incarcerated persons. A French study demonstrated that prevalence for prison population was above the rate for an extramural group (Combalbert et al., 2018). A recent German study that used cognitive screening tools indicated that up to 40% of study participants from closed detention over 60 years of age have cognitive impairments (Verhülsdonk et al., 2021a). Studies exist that point to an increased presence of risk factors for the development of cognitive deficits and dementia in older prisoners (see Brooke et al., 2020 for review) Physical, cognitive, and social inactivity as well as an unbalanced diet seem to be the major influencing factors (Christodoulou, 2012). However, with regard to the prevention and treatment of cognitive dysfunctions in older prisoners, studies are lacking.

One approach to prevent and treat cognitive dysfunctions in older people that has attracted increased interest during the last decade is cognitive training. This intervention refers to the targeted training of cognitive functions (e.g., memory, attention) using paper-and-pencil and/or computerized tasks as well as psychoeducation (Clare and Woods, 2004; Kalbe et al., 2010; Bahar-Fuchs et al., 2013). The goal is to improve, stabilize, or restore cognitive functioning. Meta-analyses demonstrate the efficacy of cognitive training in older cognitively unimpaired individuals (Lampit et al., 2014; Chiu et al., 2017; Hill et al., 2017; Gavelin et al., 2020; Yun and Ryu, 2022). Also, in individuals with mild cognitive impairment (MCI) as a possible dementia pre-state, significant improvements in cognitive (e.g., global cognition, memory, attention, executive functions, working memory) and non-cognitive outcomes (e.g., depression, anxiety) have been shown (Gates et al., 2011; Hill et al., 2017; Sherman et al., 2017, 2020; Ge et al., 2018).

Within the context of the prison system, there has been a lack of studies evaluating cognitive training - despite the obvious positive effects on individuals with and without cognitive impairments. Since cognitive impairments are associated with high health economic consequences (Olesen et al., 2012) there should be a societal interest in targeted prevention and therapy. This is increasingly relevant, especially against the backdrop of demographic change and the associated rise in people suffering from dementia (Bickel, 2018). For the prison setting, the relevance of treatment increases even more because of the association of cognitive impairment and adverse health and crime outcomes (Ahalt et al., 2018). Thus, it impacts on prisoners’ release planning and resocialization.

Against this background we conducted an intervention study in which we conducted a cognitive training program (“NEUROvitalis Prison”). In order to gain an in-depth understanding of the effects of the intervention and perceptions on different levels (e.g., end-users and service providers), we conducted a mixed methods study that included a one-arm feasibility trial with quantitative analyses as well as qualitative methods in form of interviews; the latter of which that is described in the following.

### 1.2 Program description of the cognitive training program

#### 1.2.1 NEUROvitalis

The multidomain cognitive training program NEUROvitalis (Baller et al., 2020) comprises 12 structured manual-based group sessions à 90 min. Each session is characterized by a repeating sequence and includes psychoeducation, group exercises, and individual paper-and pencil-exercises. Psychoeducation takes about 20 min in each session; cognitive exercises take about 70 min. NEUROvitalis was developed by neuropsychologists and gerontologists and has been conducted in studies with, e.g., cognitively healthy older people (Rahe et al., 2015; Roheger et al., 2019), and people with Parkinson’s disease with and without mild cognitive impairment (Petrelli et al., 2014; Kalbe et al., 2020).

#### 1.2.2 Modification of the cognitive training for the prison setting–NEUROvitalis PRISON

Each session has a specific theme (see Table 1), which then guides the psychoeducation as well as exercises within each session. The cognitive training program was adjusted for application in prison settings. Therefore, the psychoeducation part was adapted to the conditions of a closed prison ward (e.g., management of risk and protective factors). Further modification will be made after the pilot study is completed.

**Table 1:** Overview of the topics of the NEUROvitalis PRISON sessions.

Session 1	Mental performance: impairments and training
Session 2	The significance of attention
Session 3	How does the memory work?
Session 4	Working memory
Session 5	Memory and language
Session 6	Memory strategies I: systematic repetition and clustering
Session 7	Memory strategies II: memorizing through different sensory channels and story technique
Session 8	Memory for names and faces
Session 9	Remembering appointments and tasks
Session 10	Understanding and remembering texts
Session 11	Planning and problem solving
Session 12	Risk and protective factors

NEUROvitalis PRISON was offered in its pilot form in a group setting during a 12-week period (09/21–12/21 and 06/22–09/22) in two prisons that contain specific older offenders departments in North-Rhine Westphalia, Germany. Within the prison context, a group size of 4 to 6 participants was perceived as appropriate. The training was free of charge and participation was voluntary. In addition, participants received further exercise material, so called `homework’ - consisting of paper- and pencil-exercises form the standardized program ‘NEUROvitalis HOME’ (Baller et al., 2018). It was conducted by external trainers, a female gerontologist with support of a student (medicine or psychology, respectively).

In order to obtain the most realistic representation possible of people aged 50 years and older in the closed prison system, broad inclusion criteria were defined. The inclusion and exclusion criteria are outlined in Table 2.

**Table 2:** Inclusion and exclusion criteria NEUROvitalis PRISON.

Inclusion criteria	Exclusion criteria
Offenders housed in the closed units for older prisoners Male gender Age: 50 years and older Native language German or sufficient language skills to actively participate in the cognitive training program as well as in the neuropsychological examinations	Physical disability or illness that counteracts study participation (e.g., blindness, deafness). Cognitive impairment to the extent of dementia with lack of capacity to consent (operationalized via the DemTect ≤8 points; Kalbe et al., 2004) Severe psychiatric symptoms (i.e., major depression, psychosis, delusions, hallucinations) that counteracts study participation Known intellectual disability

All interested prisoners who met these criteria and provided written consent for study participation were enrolled in NEUROvitalis PRISON. Since the pilot program was planned in two departments for older male prisoners, additional staff of the prisons was not required. However, in consultation with the participating correctional facilities, it became apparent that the staff would very much like to be involved in the administrative part of the implementation of the cognitive training in order to ensure sustainable implementation post cognitive training. Social services providers of the prison assisted in communicating about the training offer, recruitment and logistical facilitation of the cognitive training.

The project was carried out in only two specific departments for older prisoners; 19 prisoners initially joined the cognitive training; 18 completed the training and one person dropped out due to language deficits. In total, 14 prisoners of the older offenders departments declined to participate.

### 1.3 Objective

The overall objective of the project was to evaluate the feasibility of the NEUROvitalis PRISON program within the closed correctional facility from both the perspectives of the programs’ end-users (older prisoners) and the prison staff post training. In this paper, we report on the qualitative outcomes only. The used neuropsychological instruments, cognitive performances (pre and post- outcomes) and feasibility outcomes are published elsewhere (Verhuelsdonk et al., 2023).

With this study we aim to gain in-depth multiperspective understanding regarding (1) the experiences with the new cognitive training program NEUROvitalis PRISON, and (2) the perceived changes induced by the training.

## 2 Methods

### 2.1 Study design and ethical procedure

An explorative qualitative study design was applied. It was approved by the ethics committee of the Medical Faculty of the University of Cologne at 25-08-2020 (ID 19–1,664) and registered in the in the German Clinical Trials Register (DRKS; ID: DRKS00020227). Relevant national and European data protection regulations were obeyed during data collection. The COREQ guideline was followed (Tong et al., 2008). Participants’ anonymity and confidentiality were protected at all times. Written informed consent was obtained from all participants included in the study. No study compensation was offered.

### 2.2 Setting and sampling of the participating groups

Individual semistructured interviews were conducted. They were conducted in both facilities where the NEUROvitalis PRISION training was held. Beforehand, data collection had to be scheduled and authorized by the head of the closed correctional facility management.

In order to gain a multi-perspective understanding, purposive sampling strategies were applied (Flick, 2009). Two groups participated in the face-to-face interviews: (1) the program’s end-users, being older male prisoners, and (2) the prison staff, employed as prison warder, social prison staff members, or psychologist (Table 3).

**Table 3:** Sociodemographic data of the participating prisoners.

	*N*	Min	Max	*M*	SD
Age	18	55	76	65.95	5.56
Education (school + vocational training)	17	0	17.00	6.71	5.62
Sentence (in years)**	15	3.00	15.00	7.28	3.70
Duration of current incarceration at time of the Interview (in years)	18	0.83	52.00	6.82	11.74

Minimum (Min), Maximum (Max), mean (M) and Standard deviation (SD). **prisoners with life-long sentences (and thus, no concrete number of years) were not included.

#### 2.2.1 Participating group 1

For the program’s end-users, comprehensive sampling was applied (Gray, 2009). If all end-users are willing to participate, the sample size can include maximum 18 prisoners. It is considered helpful for studies that focus on a specific organization or people or specific, uncommon characteristics and a population size that is somewhat small and therefore allows to include the entire group (Gray, 2009). In our case this target group were older German male prisoners who had previously enrolled in NEUROvitalis PRISION.

#### 2.2.2 Participating group 2

For the prison staff, maximum variation sampling was applied that aims at apprehending and describing a wide range of variations (Patton, 2002; Gray, 2009). Our aim was to explore at least the perspective of the different staff members of the two prisons.

### 2.3 Data collection

The semistructured interview guideline was developed by the interdisciplinary team of gerontologists and psychologists (Annex 2). It contained two main topics: participants’ individual program experiences and program fit as well as perceived modifications due to the program. Interviews were conducted face-to-face by the first author within 14 days after the training was finalized. During data collection, there were no other people in the room besides the interviewer and the interviewee; notes were taken meanwhile.

### 2.4 Data analyses

Data Analyses was conducted from January till December 2022. First, audiotaped data were fully transcribed externally by an independent person that considered the standards for social research (Dresing and Pehl, 2018). Second, transcripts were anonymized by the research team, each transcript was labeled with an interview ID number and all possible identifiers were detached (e.g., names of participant, profession or study sites). Third, two researchers (third and last author) coded the transcripts independently by applying deductive-inductive content analysis (Graneheim and Lundman, 2004; Rädiker and Kuckartz, 2019) supported by the software MAXQDA 2020. Fourth, based on the topics of the interview guideline, a first code tree was developed deductively. Fifth, the entire material, emerging inductive themes and sub themes, was discussed among the two researchers with the aim of achieving a profound understanding of the material. Themes and sub themes were condensed. The entire process was critically accompanied by discussions among the coders, which continued until a consensus about how to group the findings was reached, whereupon a final coding system was agreed on. Finally, illustrative quotes were extracted and translated in English for this manuscript.

## 3 Results

### 3.1 Sample characteristics

In total, 23 persons participated in the study: 18 prisoners and four staff members (two correctional officers, one social worker and one psychologist; Table 2). Nineteen prisoners completed the training; one person dropped-out due to hospitalization.

The total interview material comprised 290 min. Interviews ranged between 4 and 35 min. Out of the material 1.183 quotes two categories, six major themes and 10 subthemes emerged (Annex 3). Exploration of the participants’ individual program experiences concerning the program fit and perceived changes induced by the program.

Data will be presented within two categories: (1) experiences concerning the program fit and (2) perceived changes induced by the program. Within the following “*P”* displays a quote from the prisoner and “S” from the staff.

#### 3.1.1 Category 1–exploring the actual program fit

Within the first category four themes emerged: program understanding, program fidelity, conceptual building blocks, and overall program evaluation.

##### 3.1.1.1 Theme 1–program understanding

Before the cognitive training started, most participants were not entirely clear about the goals or benefits, however, all of them were open minded. Some of the participants perceived the cognitive training as a great opportunity to *“get out of the cell”* [P4] and some came with low or *“no expectations”* [P7]. One person even felt confronted by the offer: *“Machine broken, old man? You cannot kink an old tree! But I was pleasantly surprised”* [P4]. One participant anticipated that the cognitive training should support cognitive vitality.

*“…the reason that I signed up for it was preceded by the insight that you forget certain things in everyday life and then I questioned myself. I realized that I didn't have any strategies to get around that to change.” [P6].*

The prison staff also perceived that the objective of the cognitive training was not entirely clear to prisoners. *“I think at the beginning the participants could not really imagine what exactly awaited them and there were somehow a few concerns or fears expressed in advance”* [S1]. Especially the issue of *“passing a test”* [S1] was perceived as intimidating.

##### 3.1.1.2 Theme 2–program fidelity

This theme is composed of three sub themes: adherence, compliance, and attitude toward training providers.

The prisoners said that it was important to them that they received constructive feedback. Most important was that they perceived that cognitive training might be important for them, especially in this setting. It was also important that the offer was free of charge and an opt-out option was given: *“…that I could stop at any time, but that would have been nonsense because that was interesting for us”* [P9]. Some of them perceived the homework as a positive competition among themselves. The program adherence was high for various reasons. The content was perceived as interesting, especially the lectures about dementia. Also, some of them also had familiar experiences with dementia or perceived themselves that their cognitive performance had decreased in the last years.

The prison staff mentioned that the participation was, compared to other offers, very constant. *“All participants really did the training from start to finish. I think two missed one appointment, each due to illness” [S1].* They explained this compliance with the concept of the program and the competence of the trainers. Prisoners showed an intrinsic motivation, were right on time and even did their homework without any pressure. The staff mentioned that participants were content that the external professional trainers were coming; they felt appreciated due to the fact that someone was truly investing in them as a person. Only one person was not satisfied, but continued the training.

*“He said he simply couldn't do anything with such a group program and didn't see any profit for himself either.”* [S2].

For the prisoners the attitude of the trainer was crucial. They appreciated that they were warmly welcomed every time and that the trainer was empathic and enduring. It was highlighted: *“You gave us the opportunity to work on it and you explained your case well”* [P9]. The training was fostering their self-esteem.

*“I was actually surprised that you did the work. We don't deserve it.” [P9].*

##### 3.1.1.3 Theme 3–conceptual building blocks

Two sub-themes emerged: practical application and content.

The practical application contained the voluntary offer base.

*“If one is offered something, then one can think about it freely, would I like to participate in it, one is forced to do nothing, so why not?* [P7].

Breaks were generally perceived as important. However, one participant mentioned: *“The smoking break got on my nerves a little bit. You should be able to control yourself for 90 min…valuable time goes down the drain.”* [P4]. Also, the duration of each session being 1 h and a half, was perceived as satisfying and appropriate. With respect to the group composition, it was perceived as appropriate and also the group size of five to six prisoners was observed as helpful for such type of cognitive training. The frequency, being one time a week, was sufficient for most participants. *“For my taste, the timing was appropriate and did not overwhelm anyone”* [P8]. However, one participant was very ambiguous: *“I could have done this every day, but that is impossible”* [P9].

Concerning the content, the team work options during the sessions were appreciated. *“I also found it quite good to help the others in the team”* [P4]. This was especially the case with the homework that were discussed among them. The training material that was distributed was used by all prisoners.

*“Some of the material was very good and there were also some papers that were a bit childish for me. But most were really very, very good.”* [P1].

Due to the satisfaction some of the games used were bought by the prison staff. Opposed to it one prisoner mentioned: *“I have read them all. However, after consultation I have also destroyed them. I simply did not want to see them anymore in my detention room”* [P6]. There was only one time mentioned that a material was not entirely understood. Prisoners and staff proposed that training material must be written in a recipient-friendly language.

The amount of the theoretical input of the cognitive training was differently valued. Some felt that it was sufficient, adequate and that they *“could also relate (to it)”* [P1].

*"It was understandable. It was supposed to be about - let's say –not totally flattening out here, yes."* [P2].

However, some felt it was sometimes too much theory or some issues were difficult to follow. In this case it was mentioned that it would be good to use a more interactive approach.

Divergent to their experiences in school as a child, most prisoners appreciated their homework. The staff recognized that *“for the time between the sessions,* [prisoners] *have always been working on it”* [S2]. However, some prisoners felt “*there could have been a bit more sometimes”* [P6] and others perceived it as challenging, especially the mathematical parts. Some prisoners did their homework together and helped each other with it or discussed the different solution options. It was regretted that when starting a new session, it was not given sufficient attention to the homework. They would like to have individual feedback on their homework.

For most participants, the interactive parts with the different games and guesswork were the highlight of each session. They felt that the balance and the mix of different interactive games, especially the games focusing on attention, were *“brilliant”* [P4]. Some perceived that doing practical exercise was as important as theory.

*“Yes, it was very informative. There were many different aspects somehow, different approaches, how you can improve one or the other, if you have difficulties…” [P7].*

It was perceived as having fun and doing some mental exercises. However, one participant stated:

*“I didn't have that with the games anyway, that's not my thing”* and felt *“unchallenged.”* [P7].

##### 3.1.1.4 Theme 4–overall program evaluation

The program evaluation was observed from two perspectives: (1) individual program evaluation of the participant, and (2) third party evaluation of the staff members. Three sub themes emerged: group dynamic, training atmosphere and summarizing evaluation.

Most of them felt that the group dynamic was very good and they felt accommodated. The staff observed *“…it was totally super for me and also to see the guys in the group community and how they have spurred each other on against each other”* [S1]. They also mentioned that both groups worked really well. According to their role as staff in the prison they mentioned *“however, we also had to make sure that the groups fit together”* [S2].

*“It was also quite noticeable that the groups have developed over time. In the beginning, there was a lot more restraint, even among the participants themselves.” [S1].*

The training atmosphere was perceived in general as welcoming and stimulating. However, one person felt annoyed by one person that did not understand well due to German language weaknesses and due to the fact that this person was interrupting and talking by himself permanently.

Overall, all participants were satisfied with the cognitive training offer. Some of them mentioned that they see a profit and would like to repeat it as if it was *“the brain is loosening up” [G1].* Only one person had concerns as it was too short and not intensive enough. Also, the staff felt that the cognitive training was important for the prisoners.

*“I found overall very coherent things and, so let's say, this interactions with you, and also with the prisoners then, for example with the fellow prisoners, absolutely, that was already good, I must say.” [P6].*

*“It went surprisingly well. But I actually think that it was also this mixture of group exercises and information transfer, because that was also very well received. I think the mixture was it.” [S1].*

#### 3.1.2 Category 2–Perceived changes induced by the cognitive training

Following two themes emerged: application of new strategies and self-monitoring.

##### 3.1.2.1 Theme 5–application of new strategies

The results are very diverse. Some did not apply any new strategies. Others experienced that they apply especially some of the memory issues and that the cognitive training itself opened their mind that they have to invest in their cognitive vitality. Some mentioned that they become more attentive and try to integrate some issues in their daily routine, e.g., diary writing, using a calendar. One person mentioned that the cognitive training has improved his concentration and another highlighted that he could better structure his day using ‘Post Its’ for important issues. One noticed that after the training his short-term-memory and his memory about names augmented.

*“I kept it and I also did that and I actually found that quite good. The exercises fit wonderfully.” [P6].*

After the cognitive training, the staff perceived that the mood in the group and department transformed. They once again become more open minded. In individual participants they even observed that they were a bit more awake and attentive again or perceived their environment more consciously again and interacted with each other differently.

*I think it has also improved their skills once again. I don't know whether this is necessarily measurable with our instruments, but I think the guys have definitely benefited from it. [S1].*

##### 3.1.2.2 Theme 6–self-monitoring

In terms of monitoring themselves following three subthemes emerged: cognitive perception, perception toward getting older, and self-perception to stay healthy.

The participants monitored themselves very differently. Some participants felt a *“bit frustrated”* [P3].

*“I still have problems when I have to repeat words or remember numbers.*

*If there are more than four, then it becomes problematic. I do not manage.“* [P3].

Some perceived positive changes induced by the training.

*“… the last time at the neurologist's office and I was able to answer 30 percent of the questions immediately, which I never knew before.”* [P1].

It was mentioned that with a little bit of self-discipline and these exercises one may remain mentally more flexible.

Toward the reflection of getting older, it was perceived by some participants to invest in their cognitive health and to perceive their weaknesses. Some felt that the age becomes noticeable in connection with physical fitness and mental fitness, because one is a bit mentally tired in the afternoon after work.

*“I don't necessarily notice any dementia or anything like that in myself yet. But in some things, it's already like that, so a few things there. So that's already important to be sensitive.”* [P3].

*“I have now read through the whole thing again from the beginning. Now I want to try to implement these possibilities that are there, which I now see as important for me personally.” [P6].*

The importance of staying healthy in prison increased for some participants. Nevertheless, some felt like *“old little trees cannot be bent”* [P4] and they did not feel like changing their habits. Conversely, other prisoners structure their lives very strongly in order to prevent depression or to feel bored.

*“Playing chess regularly, solving Sudoku regularly, intelligence questions, that's defending dementia, after all. So, do it!” [P4].*

## 4 Discussion

The qualitative approach provided a rich understanding of the experiences of the cognitive training program and the perceived changes it brought about at the individual and system level. To the best of our knowledge, this is the first study that evaluates the implementation of a cognitive training for older inmates. We were able to include two perceptions that of (1) the prisoners as program end-users, and (2) of prison staff members.

Both, the prisoners as well as the staff highlighted the cognitive training program as a very positive and stimulating experience. Furthermore, the importance of the training in terms of increased self- and knowledge-transfer to everyday life was also pronounced.

### 4.1 Program experiences

Both, the prisoners themselves as participants of the workshops and the prison staff members, stated that they did not have any concrete ideas or expectations beforehand. Unfortunately, there are no comparable data to which the results presented here can be related within the prison setting. This type of training, including group exercises and psychoeducation, was completely new for the participants. This highlights the exploratory nature of the analysis presented here. As described by Borglin (2015) for interviews with healthcare professionals, exploratory interviews provide insights into contextual factors and barriers that can be used for adjustments of program, practical application or materials.

The program participation was offered on a voluntary base without any charges and independently from the respective penalty and, thus, a low-threshold offer. In the beginning, prisoners’ decision to participate in the training program was based on the possibility to leave their cells and to have a meaningful occupation. This aligns with findings from pointing to the possibility of escapism from cell using voluntary educational offers (Panitsides and Moussiou, 2019). The analysis of qualitative interviews of older inmates emphasized the importance of evocative occupation for one’s own identity (Haesen et al., 2018). Cognitive training can be seen as a measure to pursue an activity that is experienced as meaningful and that can also contribute to self-esteem. They state that an occupation, regardless of whether it is work or leisure time activity, is identity-affirming and self-esteem-sustaining and, thus, has a value far above that of ordinary occupation. A special importance seems to be devoted to the program providers of the new program and their working approach or inner attitude. It was perceived as open-minded and participatory, which might be decisive for the training atmosphere, which is in line with other findings from other prison studies with different age groups (Halimi et al., 2017).

Both, the participants and staff members conveyed that the training was perceived as very important, especially with regard to life circumstances in prison setting. This is underlined by the fact that incarceration is associated with an increased risk of early cognitive decline in older people (Du Toit and Ng, 2022). At this point, the necessity of bringing in experts from the outside is also pointed out once again.

For the prisoners, this circumstance may contribute to the high level of motivation that has also been reported for educational opportunities in this setting (e.g., Panitsides and Moussiou, 2019). Based on qualitative interviews, Novisky (2018) concludes that older prisoners in particular have the desire and the effort to acquire medical knowledge themselves. They also point to the increased interest of the correctional institutions in providing age-appropriate care for this population. Panitsides and Moussiou (2019) also highlight the aspect of empowerment due to the participation and the impact on everyday-life in prison.

As described for other groups of subjects, the NEUROvitalis program seems to be well accepted by the group of older prisoners as well. Consistent with studies concerning NV Training with clinical populations (e.g., Kalbe et al., 2020), in our case the motivation is also reflected in a high number of attended trainings and a zero dropout rate. Also, the exercise sessions which were assigned as homework were well received. It was utilized for their own examination of the topics and intensification of the exercise. Especially the transfer-to-everyday-life-strategies and the use of homework was also described as beneficial in another study that explored cognitive training in an extramural population (Belleville et al., 2006). This result underlines the practicability of the used program.

The content defined in the original program development as well as the structure of the overall program and the time frame (Baller et al., 2020) were also considered appropriate by the participants in our study. Moreover, the division into 12 single sessions per week was compatible with the organizational processes in the prison department. Thus, the modification of the original program with two sessions per week seems to fit well in the correctional setting. Training of different cognitive domains such as executive functions, attention, and memory as a multidomain approach has been found to be beneficial in other studies with other cognitive training programs in older people with MCI (e.g., Granland et al., 2022).

The practicability of such a group program for training cognitive functions experienced in the context of our own project hardly differs from the experiences made in other settings: Woolf et al. (2021) also describe very positive experiences in the clinical setting, and here, too, the combination of training content and psychoeducation was very well received. However, those authors promoted an adaptation of the training materials in the sense of a simplification of the language after the first project phase. Although we modified the program, some of the participants criticized that some aspects were presented too complexly. Therefore, we encourage an adaptation of the training materials to simplify the language for future implementation attempts.

Cognitive training may also contribute to counteracting the depressive symptoms, were the depressed participants showed a high level of acceptance (Hammar et al., 2022), also often described for inmates (Verhülsdonk et al., 2021b). Thus, we indorse that the implementation of such a resource-activating program may have a positive effect on the prisoners, as the tendency to ruminate in depressed patients decreases as well.

### 4.2 Perceived changes induced by the cognitive training program

Participants report that they benefited from taking part in the program. Strategies to enhance memory functioning were transferred into everyday life, in some case attention and concentration were described to be strengthened. This appears to be important, since the group of older prisoners is exposed to an increased risk of cognitive dysfunctions and even dementia (Forsyth et al., 2020). Programs that promote these performance-improving abilities are therefore also important for coping with everyday life in the community. Chamberlain and Dening (2020) point out the importance of preventive measures, especially in the already stressful prison setting. It is also emphasized that older prisoners are health-conscious and that here, too, people are concerned with their own aging process. Especially the possibility of transferring learned contents into one’s own everyday life seems to be a significant aspect, which has been emphasized in other studies with extramural populations (Woolf et al., 2021; Granland et al., 2022). This issue and the feeling of developing individual difficulties with increasing age, for example with memory was also reported by some of the participants in our study.

Prison staff members also perceived changes in the atmosphere in the unit post cognitive training. As described by Mukeredzi (2021) an involvement of prisoners in educational offers is associated with a decrease in crime and increase of better atmosphere. Therefore, implementation of offers like the cognitive training program should be given increased consideration.

### 4.3 Overall relevance of the program implementation into the prison setting

In general, the principle of equivalence, which is anchored at least in the German Criminal Code, also states that incarcerated persons are entitled to the same benefits as those for the treatment of illnesses, but also to preventive measures to avoid illnesses. The development of age-specific health and support services not only contributes to medical care in accordance with the principle of equivalence, but also contributes to successful resocialization and can help to reduce costs due to follow-up illness. A particular relevance arises from the fact that the group of older prisoners is growing and there is a lack of strategies to meet the special needs of this group. Based on semi-structured interviews with both prisoners and staff members, Senior et al. (2015) conclude that there is a lack of age-specific services including day-time activities. They also postulate a written older-prisoners-policy for the institutions. Seaward et al. (2021) even call it an “agequake,” because the increasing number of older offenders in correctional settings and the care they receive poses challenges. The United Nations have defined this group as a particularly vulnerable group (Atabay and Atabay, 2009). Furthermore, the socio-economic importance of measures that help prisoners to achieve a positive identity and thus counteract the risk of mental illness and of recidivism is emphasized (Haesen et al., 2018; Mukeredzi, 2021). In summary, this underlines the relevance of the topic as well as the need to target the group of older inmates in everyday prison life. For that purpose, our feasibility study took the first step in this direction.

### 4.4 Strengths, limitations, and lessons learned

To our best knowledge, this is the first cognitive training trial in the prison setting. It was provided by external professional trainers which might have been fostering the motivation to join and complete the training. Thus, depending on personal resources of the institutions, as a next step a train-trainer-program could be offered or external trainers could be engaged for the implementation.

Our data collection took place immediately after the cognitive training. It would have been enlightening to explore the sustainability of the cognitive training (e.g., 6 months post intervention). However, due to COVID restrictions were not able to conduct a follow-up data collection.

#### 4.4.1 Transferability and generalization

Due to this study, we gained a sound understanding of the optimization needs. In the future, the cognitive training should be offered to a larger group of prisoners within different prisons in different national countries in Germany. Further, it might be also tested if the cognitive training is transferable to other nationalities.

Only male prisoners participated. Thus, our results cannot be generalized in terms of gender. How the implementation of cognitive training will be is experienced and evaluated by younger or female prisoners still needs to be investigated.

#### 4.4.2 Study design

The qualitative design was appropriate to gain a multi-perspective and comprehensive understanding of experiences and perceived alterations and the practicability of the cognitive training. However, due to the restricted regulations within the setting and the study type (explorative cognitive training study), we were not able to enter in iterative optimization loops and follow-up interviews. Besides, conducting quantitative analyses may underbuilt the outcomes of the end-users.

#### 4.4.3 Sampling

In order to gain a sound understanding, purposive sampling strategies were applied for both end-users and prison staff. In our case the qualitative samples were predetermined (Flick, 2009), therefore seeking for saturation was impossible.

Concerning the end-users, we were lucky to apply a comprehensive (or total population) sampling (Gray, 2009), which is perceived as “ideal” for further analyzing, differentiating, and perhaps testing (Flick, 2009). Moreover, it may augment confidence in the validity of the outcomes of this very specific cognitive training, as it covers every case in a given population. Therefore, it may diminish the risk of missing valuable insights. Interestingly, all participants of the study were willingly to participate in the “outcome-Interview” only one person became sick and could not participate for that reason. This circumstance shows their intrinsic motivation and satisfaction with the program. However, comprehensive sampling is only applicable to very specific studies because it requires the targeted population to be small and have uncommon characteristics (Gray, 2009). Therefore, our outcomes contain very limited potential for generalizability.

It was possible to gain a multi-perspective understanding by gathering the perception of the prison staff maximum variation sampling was applied (Patton, 2002; Gray, 2009), which enabled us to capture all variations of a phenomenon and explore detailed insights about each variation (Patton, 2002). However, the participation of the staff was determined by their schedule and availability at the moment of data collection. So, the sample size is very small. It would have been interesting to collect more perspectives.

## 5 Conclusion

Our exploratory study highlights the need for the implementation and optimization of age-specific educational offers in the correctional setting. The conducted cognitive training program was highly appreciated and experienced as very positive by both the programs’ end-users (prisoners) and members of the prison staff. The program’s content as well as its structure with individual training approaches, group exercises and psychoeducation were experienced as beneficial. The majority of the end-users were in favor of the continuation and the meaningfulness of such an offer. These statements were supported by the regular participation as well as by the high acceptance and self-initiated implementation of the “homework exercises.” Thus, the cognitive training program seems to be an adequate offer for older prisoners in terms of dementia prevention and cognitive training.

## Data availability statement

The datasets presented in this article are not readily available because according to the participant informed consent form, the interview data are not available for scientific use by anyone other than the project group members. Requests to access the datasets should be directed to sandra.verhuelsdonk@lvr.de.

## Ethics statement

The studies involving humans were approved by Medical Faculty of the University of Cologne at 25-08-2020 (ID 19–1,664). The studies were conducted in accordance with the local legislation and institutional requirements. The participants provided their written informed consent to participate in this study.

## Author contributions

SV: Conceptualization, Investigation, Methodology, Supervision, Validation, Writing – original draft. A-KF: Conceptualization, Investigation, Methodology, Writing – review & editing. CH: Data curation, Formal analysis, Investigation, Project administration, Writing – review & editing. CB: Data curation, Formal analysis, Investigation, Project administration, Writing – review & editing. JC: Methodology, Project administration, Supervision, Validation, Writing – review & editing. EK: Conceptualization, Methodology, Project administration, Supervision, Validation, Writing – review & editing. TK: Data curation, Formal analysis, Methodology, Supervision, Validation, Writing – original draft, Writing – review & editing.
