# Supplementary material for: Cognitive training for older prisoners: a qualitative analysis of prisoners’ and staff members’ perceptions
Source: Front Aging Neurosci. 2024 Jan 8;15:1332136. doi: 10.3389/fnagi.2023.1332136 (PMC10800784; doi:10.3389/fnagi.2023.1332136)

**Supplementary Material**

**ANNEX 1**

*Interview questions - prisoners*

How have you experienced the past 12 weeks with regard to the implementation of the cognitive training? What are your general impressions?

- How did you find the frequency of the training? Should it be more frequent or less frequent? How would you rate the duration of a single session? Should the sessions be longer or shorter?

- How did you feel about the group constellations?

- How well did you like the concept, the topics and content, and the materials for the exercises? Where do you see suggestions for improvement? Which topics did you miss or what would you have liked to talk about in addition?

- In addition to the group meetings, you were encouraged to do further exercises for your mental performance. How were you able to implement this into your everyday life? What did you do? If no further exercises were carried out: What reasons can be given for this?

- Should a cognitive training program be established in the future as well? What speaks for it, what speaks against it? How motivated would you be to participate in a cognitive training program in the group or to train independently in the future?

- To what extent and at what times would such an offering be most feasible? What group size would be ideal?

- Do you have any other comments or anything you would like to add?

*Interview questions for prison staff members*

- How have you experienced the last 12 weeks with regard to the implementation of cognitive training? What are your general impressions?

- How high do you estimate your additional workload during the last 12 weeks due to the cognitive training?

- What is your assessment: is such a training program implementable in the daily routine of a department for life-aged detainees? What are the arguments in favor? What are the arguments against it?

- What is your assessment of the implementation feasibility as of today, after completion of the 12-week training as part of the study?

- How did you find the frequency of the training for participants? Should it be more frequent or less frequent? How would you rate the duration of a single session? Should the sessions be longer or shorter

- What was your impression of the participants before, after, and between sessions? What was the participants' reaction to the cognitive training?- Do you have any other comments ?


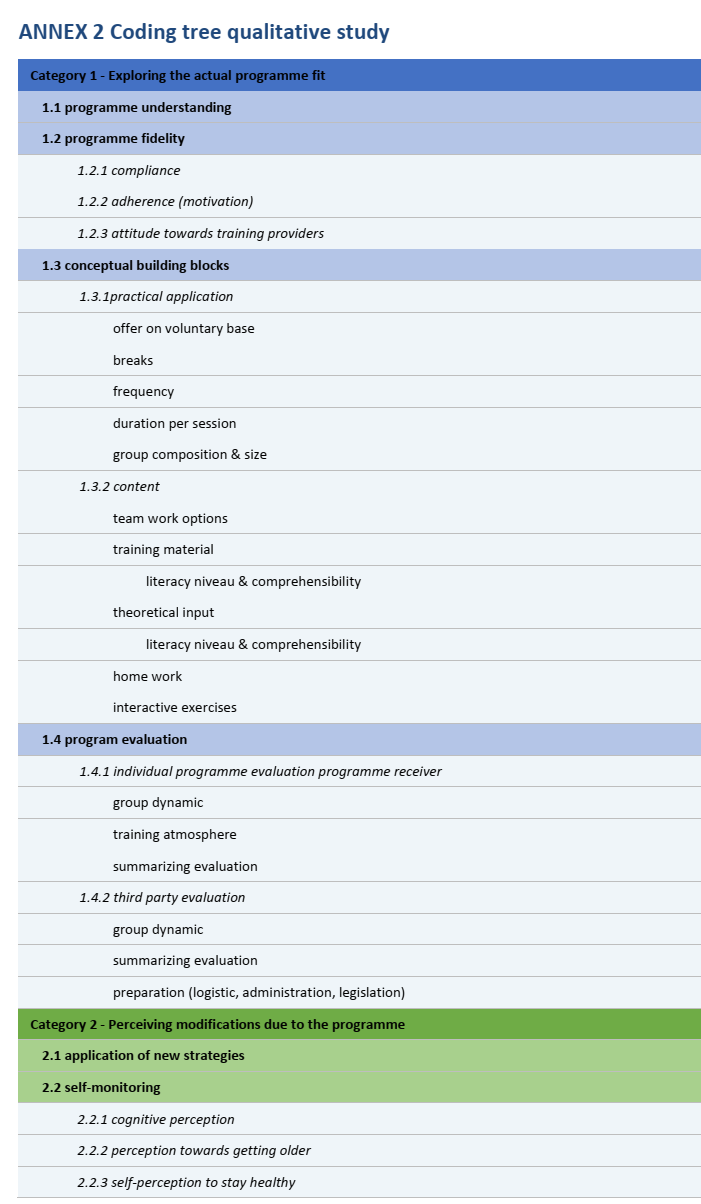

Supplement: Supplementary file 1 [file Data_Sheet_1.docx]
